# A plea for a more dialectical relationship between personal and subpersonal levels of analysis

**DOI:** 10.3389/fpsyg.2014.01165

**Published:** 2014-10-10

**Authors:** Michele Di Francesco, Massimo Marraffa

**Affiliations:** ^1^Institute for Advanced Study IUSSPavia, Italy; ^2^Department of Philosophy, Communication and Visual Arts, University of Rome ‘Roma Tre’Rome, Italy

**Keywords:** attachment, dynamic psychology, folk psychology, interface problem, mark of the cognitive, motivation, subpersonal level, the unconscious

Bermúdez (2005) termed “the interface problem” the question of clarifying how typical subpersonal explanations in cognitive sciences, whatever is their specific form, are related to folk psychology. In this opinion article we will approach the interface problem from a specific angle, i.e., the relationship between consciousness and the unconscious as it has taken shape within cognitive sciences.

Our starting point is the contrast between the cognitive unconscious and the Freudian one. If examined from an orthodox cognitivist point of view, psychoanalysis turns out to be a brilliant but failed attempt to build a genuine subpersonal psychology. Freud aims to go beyond the psychology of his times, which is a psychology of consciousness; his theory of the unconscious is, therefore, *programmatically* against a “consciousness-centric” mentalistic framework. The problem is that, *as a matter of fact*, Freud failed to extricate himself from that framework. Like many psychoanalytic ideas, the Freudian unconscious is just an enlargement, or extension, of a psychology—folk psychology—hinged on the idea of a person who is able to have conscious mental experiences.

According to a number of philosophers this extension of our ordinary psychological conception of mind is a strength of psychoanalytic theory. In this perspective, the grounds for psychoanalysis “lie in its offering a unified explanation for phenomena (dreaming, psychopathology, mental conflict, sexuality, and so on) that commonsense psychology is unable, or poorly equipped, to explain” (Gardner, 1999, p. 684). This approach has been taken as the basis of a defense of psychoanalysis against well-known epistemological objections: like folk-psychological explanations, psychoanalytic explanations should be exempt from the epistemological and methodological requirements of experimental science (Manson, 2003, p. 179). Donald Davidson is one the referents of this conception of psychoanalysis. On his view the personal level is autonomous and different from the subpersonal one, and is to be studied by means of different methods: you need *hermeneutics*, not the quest for natural laws. That is, the folk-psychological explaining is here viewed as an interpretive activity aimed to give sense to behavior—to “rationalize” it. Accordingly, when one runs across such a “pathology” of reason as self-deception, the personal psychology framework is not to be given up in favor of the subpersonal one, but rather it must be enlarged or extended so that one can find somewhere else the rationality set out by the principle of charity. In this vein, the psychoanalytic partitioning the mind is seen as a metaphoric device to coherently describe within the personal-level explanatory framework a phenomenon (self-deception) that otherwise would be uninterpretable (Davidson, 1982).

This attempt to abandon Freud's positivistic naturalism and reconstruct psychoanalysis on hermeneutic grounds has a very long story. In the 1970s an influent version of this project was initiated by a number of psychoanalysts of Rapaport's school; especially George Klein and, close to his ideas, Roy Schafer. According to these psychoanalysts the “biologistic” Freud is no longer defensible, and the whole Freudian metapsychology is to be declared waned owing to its association to the drive-discharge theory. By contrast, we have to reconsider the psychoanalytic clinical theory insofar as it rests on the intentionality of the interpretive process.

This “clinical theory versus metapsychology” argument, however, tries to regenerate psychoanalysis by renouncing to its main legacy. For Freud's hypothesis of a biological component that is constitutive of mental life is just what ensures for the psychoanalytic theory its typical content of systematic objection against the claim of self-legitimation made by rational consciousness: therefore, it is the ground of the very idea of a subpersonal-level unconscious. The Freudian hypothesis, to the extent that it views human subject as “tossed about” by its own biologicity, rules out that inner life can regain its own center in the free intentionality of consciousness. Vice versa, a psychoanalytic hermeneutics entirely aimed at insisting on the theme of meaning at the expense of the “blind” and “biological” theme of drive dynamics, runs the risk of surreptitiously reintroducing the pre-Freudian picture of the conscious subject as primary subject.

In this perspective, the hermeneutical approach to psychoanalysis is to be contrasted with the project of replacing Freud's positivistic naturalism with a neurocognitive naturalism (see, e.g., the writings of other two members of Rapaport's group: Holt, 1989; Rubinstein, 1997). Thus, a dynamic psychology that aims to develop psychoanalytic theories under the guidance of cognitive sciences fully confirms the *critical* content of Freud's theory of unconscious, i.e., its being a repertoire of tools to penetrate the self-defensive nature of self-conscious subjectivity. But here the Freudian personal-level unconscious is superseded by a level of analysis that aspires to be genuinely *subpersonal*: the information-processing level, wedged between the personal sphere of phenomenology and the subpersonal domain of neurobiological events.

However, the advantage of a dynamic psychology driven by cognitive sciences against the hermeneutical approach to psychoanalysis might turn out to be problematic. The reason lies in the convergence of two related issues: the interface problem and “the mark of the mental” problem. If we try to solve the former in a strong reductive way, the personal mind is to be defined in terms of the subpersonal mind. But then the question arises whether we can really explain when a sub-personal phenomenon deserves the title of mental without any reference to personal, folk-psychological concepts. In the case of a negative answer, the overall strategy of superseding personal with subpersonal psychology would be in danger.

Let us see in more details how the problem arises. Any bottom-up approach to cognition that rejects the primacy of the personal level should explain how the personal phenomena described by commonsense psychology in terms of conscious, deliberate, linear processes, which introduce “prescriptive or normative” concepts that “have no echo in physical theory” (Bermúdez, 2005, p. 44), are in fact a product of unconscious, automatic, parallel, sub-personal mechanisms. If that is so, the attempt of the radical naturalist to explain the genesis of personal-level psychology starting from sub-personal, unconscious mechanisms is quite demanding, since the gap between the two levels looks wide and deep.

Apparently, the radical naturalist has a simple way out: the concepts that “have no echo in physical theory” should be eliminated from scientific psychology just as it happened in the past, when scientific progress led to drop the protoscientific theories of phlogiston and caloric fluid. Commonsense psychological explanation should not be taken at its face value, but (at best) as a useful device for practical purposes. But now the mark of the cognitive problem strikes: the radical naturalist who rejects the intuitions about the mental embedded in our folk-psychological explanatory practices must offer a criterion to distinguish the sub-personal processes that are genuinely mental from those that are not. Without such a criterion, the emancipation of subpersonal from personal psychology is illusory. Yet, the task of making a principled distinction is not an easy one. For it is quite obvious that in the brain there are many unconscious, automatic, parallel mechanisms that, albeit not mental in nature, have a basic role in the existence of mentality. As Damasio (2010, p. 73) noticed, for example, certain brain regions such as the spinal cord and the cerebellum give contribution to essential brain functions, but are not essential to mind-making.

In other words, when we try to understand the relation between subpersonal and personal levels of psychological explanation, we face a dialectic between dependence and autonomy. If we consider the personal mind as completely autonomous, we fall in hermeneutics and in anti-naturalism, losing contact with the scientific development. If we adopt a strong vision of the thesis of dependency, we end up adopting eliminative or reductive approaches that are at risk of losing the mental as their own object of study, replacing it with objects that belong to different levels of analysis. That being so, the wisest strategy may be to pursue reflective equilibrium between dependence and autonomy, namely, working back and forth between the ordinary image of ourselves as self-conscious, intentional, rational agents, and the scientific conception of ourselves as biochemically-implemented computational machines, by revising these two images wherever necessary so as to pursue the regulative ideal of a coherent self-conception.

A good example of a research area in which a dialectical relationship between personal and subpersonal levels of analysis turned out to be extremely fruitful is provided by the way in which psychological constructs very close to the personal level such as motivation and attachment served as bridges between dynamic psychology and cognitive sciences.

The notions of motivation and attachment are at the core of contemporary psychodynamic theories that are fruitfully interacting with cognitive sciences (see, e.g., Fonagy et al., 2002; Lichtenberg et al., 2011). Now, the constructs of motivation and attachment can be definitely considered an advancement over the concepts that were formerly used to account for the same phenomena. But as we said, the concepts of motivation and attachment are very close to the personal level, and what is more they are not very precise. In other terms, their usefulness notwithstanding, they did not undergo that process of “fragmentation and reconfiguration” through which experimental psychology and cognitive neuroscience have put folk-psychological categories like attention or memory (Churchland, 1986, p. 365). What can be said in favor of motivation and attachment is that these concepts are more precise and work better than others.

Therefore, when the term “motivation” is defined as the whole spectrum of those factors that trigger, maintain, intensify, modulate or terminate physical activities or psychological events of any kind, we easily realize that it is a term that groups a heterogeneous bunch of factors, which are very difficult to classify (Jervis, 1993, pp. 288–289). At times such factors are to be examined one by one; but often it is useful to consider them all together under the label “motivations.” The main point here is that in any case the use of such term was a conceptual progress—e.g., over the 19th Century concept of will.

The term “attachment” too does not refer to a homogeneous and well-identifiable phenomenon; it is a “bond,” a term that is to be strictly and exclusively construed as a metaphor. There are attachment behaviors (due to different factors), and there are subjective experiences of attachment, which also can barely be grouped together and classified; but attachment in itself is an idea between the imaginative and the abstract, which originates from the extension of expressions such as “to keep attached” or “adhered.”

The epistemological moral that can be drawn from this case of dialectic interaction between personal and subpersonal levels of investigation can be concisely expressed in the following way. According to the eliminativists, history of psychology consists in a linear process through which the systematic research supersedes and goes beyond commonsense psychology—and together with it philosophical psychology. But things are more complicated. The progress of psychology is not due *only* to the elimination of the concepts (and the models and metaphors) of commonsense psychology in favor of the constructs of scientific psychology. Sometimes progress occurs because non-strictly scientific and unclear concepts are superseded by new concepts that are as much insufficiently scientific and yet more appropriate and precise (Jervis, 2011, p. 167).

Thus, there can definitely be terms of the personal-level psychology that are “unsuited *per se* for scientific or theoretical purposes” (Wilkes, 1988, p. 196). The aforementioned concept of will is a case in point. However, in other cases—like those of “motivation” and “attachment”—the ontological vagueness of a concept may be compensated by pragmatic virtues such as, e.g., the potential to increase the explanatory resources in some area of scientific psychology. In short, the eliminativist primacy of metaphysical considerations over the epistemological ones cannot be generalized: it is necessary to evaluate on a case-by-case basis.

## Conflict of interest statement

The authors declare that the research was conducted in the absence of any commercial or financial relationships that could be construed as a potential conflict of interest.
